# Structure insights of SARS-CoV-2 open state envelope protein and inhibiting through active phytochemical of ayurvedic medicinal plants from *Withania somnifera*

**DOI:** 10.1016/j.sjbs.2021.03.036

**Published:** 2021-03-18

**Authors:** Raed Abdullah Alharbi

**Affiliations:** Department of Public Health, College of Applied Medical Sciences, Majmaah University, Al Majmaah 11952, Saudi Arabia

**Keywords:** SARS-CoV-2, Protein, *Withania somnifera*, Protein Informatics, Bioinformatics, Public Health

## Abstract

Coronaviruses have been causing pandemic situations across the globe for the past two decades and the focus is on identifying suitable novel targets for antivirals and vaccine development. SARS-CoV-2 encodes a small hydrophobic envelope (E) protein that mediates envelope formation, budding, replication, and release of progeny viruses from the host. Through this study, the SARS-CoV-2 E protein is studied for its open and closed state and focused in identifying antiviral herbs used in traditional medicine practices for COVID-19 infections. In this study using computational tools, we docked the shortlisted phytochemicals with the envelope protein of the SARS-CoV-2 virus and the results hint that these compounds interact with the pore-lining residues. The molecular level understanding of the open state is considered and the active inhibitors from the phytochemicals of Ayurvedic medicinal plants from Withania somnifera. We have thus identified a potential phytochemical compound that directly binds with the pore region of the E protein and thereby blocks its channel activity. Blocking the ion channel activity of E protein is directly related to the inhibition of virus replication. The study shows encouraging results on the usage of these phytochemicals in the treatment/management of SARS-CoV-2 infection.

## 1 Introduction

The RNA virus is terrible in nature due to its tendency to cause sudden outbreak and among that, the coronavirus is highly cause for recent pandemic situations (Paital, 2020). Specifically, MERS (Middle East Respiratory Syndrome) and SARS (Severe Acute Respiratory Syndrome) are considered high-lethality viruses that trigger infections of the common cold to fatal pneumonia (Hajjar et al., 2013). In the follow-up, SARS-CoV-2 also joins the game, and the 2019 outbreak caused by the novel coronavirus (2019-nCoV) and disease referred to as COVID-19. In 2020, COVID-19 is the most recognized word because of its outbreak and has resulted in infections of up to 111 million people by February 2021 (Selvaraj et al., 2020c). The genomic architecture of SARS-CoV-2 has RNA ~ 30 kB on translation, converts to four structural proteins, sixteen non-structural proteins and ten accessory proteins. The structural proteins are Spike protein (S), Envelope protein (E), Membrane protein (M), Nucleocapsid protein (N) is playing the vital function of forming the virus protein interface to the external environment (Satarker and Nampoothiri, 2020). The S protein fuses with ACE2 (Angiotensin-Converting Enzyme 2) in the first step with the receptor binding domain (RBD) and initiates the virus host recognition mechanism. Another structural protein, N and M respectively involved in forming the nucleocapsid proteins and provide shape to the viral envelope (Tang et al., 2020). Here S interaction with M required for retaining the S in ERGIC/Golgi complex and also for integrating the new virions, M interactions with N plays stabilizing the capsid stability/assembly and M interactions with E playing the role in viral envelope formations and also in release of new virions. They are all active from entry to exit mechanism of viral pathogenesis inside the human host cell (Mukherjee et al., 2020).

Among the structural proteins, E protein is integral membrane ion channel protein, but short in length comprises 76–109 amino acids. In this, 07th to 12th amino acid is hydrophilic, followed by long hydrophobic transmembrane domain (TMD) and a long hydrophilic carboxy terminus (Bianchi et al., 2020). In E protein, the specialized structural phenomenon TMD contains, an amphipathic α-helix which oligomerize to form ion-conducting pore in membranes. In this ion channel, the ions are allowed to pass through the lipid channels in ERGIC/Golgi membranes, which results in activation of NLRP3 inflammasome and production of IL-1β (Farag et al., 2020). E protein plays imperial role in the human cell disruption, immune evasion, pathogenesis, virion structure formation and exit (Tang et al., 2020). This makes E protein as an attractive target for the drug design after the spike and main proteases (Nayarisseri et al., 2020). For identifying the inhibitors, there are several methods to find suitable drug candidates and now a days, powerful computational screening is available (Shah et al., 2020). But the intention of the work is to find the potential phytochemical compounds from natural herbs for treating SARS-CoV-2, by considering channel form of E protein. For tracking suitable inhibitors, the traditional medicinal plants are analysed and found that, compounds from *Withania somnifera* may have the ability to interact with E protein and inhibit the viral pathogenesis (Kumar et al., 2020). For this, the desired compounds from *Withania somnifera* are docked with pentamer chain E protein of SARS-CoV-2 and the results are provided. From this work, the results show that *Withania somnifera* based inhibitors are having high potential and can effectively block the critical viroporin with high specificity and affinity.

## 2 Materials and methods

### 2.1 Protein-Protein interaction analysis

The viral E protein interactions with human protein for both SARS-CoV (Pfefferle et al., 2011) and SARS-CoV-2 (Gordon et al., 2020) is analysed with CoVex protein–protein interaction prediction server. Here, each viral protein will be displayed with interacting host proteins and for this study, the E proteins interactions with host proteins, as per literature is visualized and provided (Sadegh et al., 2020, Sivakamavalli et al., 2014).

### 2.2 Multiple sequence alignment

The E protein sequence from SARS-COV (Sequence information from PDB ID: 2MM4) and SARS-CoV-2 is visualized for sequence alignment. But the NMR solved structure for E protein from SARS-CoV-2 is not solved fully (Mandala et al., 2020, Yadav et al., 2014), and so the whole sequence (Uniprot ID: P0DTC4) is taken and visualized for multiple sequence alignment using Praline sequence viewer available in https://www.ibi.vu.nl/programs/pralinewww/ (Dijkstra et al., 2019, Selvaraj et al., 2014).

### 2.3 Homology modelling

As stated above, the full length and open state of E protein has not yet solved and so, the sequence of E protein from SARS-CoV-2 (Uniprot ID: P0DTC4) is taken from uniport and searched for suitable templates using the blast server (https://blast.ncbi.nlm.nih.gov/) (Altschul et al., 1990, Fazil et al., 2012). Suitable template for building the whole proteins is chosen for multiple template-based modelling and subjected to modeller 9v7 (Beema Shafreen et al., 2014, Choudhary et al., 2020). Based on the templates, three models are generated and multiple chains are formed as homopentameric ion channel. The final protein structure is visualized for the validation server using SAVS server 3 and based on Ramachandran plot, the final protein is chosen for the study (Selvaraj et al., 2020a).

### 2.4 Protein and ligand preparation

The Modelled protein, along with SARS-CoV E protein (PDB ID: 2MM4) and SARS-CoV-2 transmembrane domain protein (PDB ID: 7K3G) from protein data bank (PDB) is taken for the study (Mandala et al., 2020). Both the modelled protein and experimentally solved structures are not accessible for immediate use of molecular modelling calculations and so the protein structures are prepared using the protein preparation wizard. Here the loops are refined, missing amino acids are refined and optimized. The final optimized structures are minimized through OPLS-FF for attaining the least energy minimized conformation up to 0.30 Å RMSD (Selvaraj et al., 2020b). Likewise, the phytocompounds from *Withania somnifera* is choosen from the literature, structures are downloaded from the pubchem database (https://pubchem.ncbi.nlm.nih.gov/) and prepared using the LigPrep module available in Maestro. Here bond orders and lengths are verified, and up to 32 conformations are formed based on the ligand rotatable bonds (Chinnasamy et al., 2020).

### 2.5 Active site prediction and molecular docking calculations

As of now, the active site information of SARS-COV-2 E protein is lacking the active site information, and so the homopentameric modelled E protein is subjected to Active site analysis using sitemap (Selvaraj et al., 2015). The regions predicted from sitemap is marked and those regions are provided as input for grids. The molecular interactions between the SARS-COV-2 E protein and prepared phytocompounds from *Withania somnifera* are performed by using the Auto Dock. Here the polar H atoms are additionally added in optimizing step by Kolman charges calculation through auto dock scripts generated by Scripps Research Institute (http://www.scripps.edu/mb/olson/doc/Autodock). For auto dock, all the calculations are performed using the default parameters and all the ligand conformations are ranked based on scoring parameters (Umesh et al., 2020). Lower energy conformations are evaluated and chosen for the molecular visualization using the Schrödinger 2D interaction visualizer.

### 2.6 Molecular dynamics simulation

The apo and docked ligand complex with best docking profiles and interactions are subjected to molecular dynamics simulations through Desmond molecular dynamics package (Selvaraj et al., 2018). The E protein is a membrane bound structure and thus, the protein–ligand complex is placed in the POPC membrane along with TIP3P water model (Loschwitz et al., 2020). The distance between the complex to edge of the orthorhombic box is measured to have 10 Å distance and minimized using steepest descent up to 2000-time steps. Temperature is changed to 310 K as per human body temperature, and pressure and pH is set to default. Pressure is maintained using the Berendsen thermostats and barostat method and NPT ensemble is performed for 12 ps followed by NVT ensemble for 24 ps (Muralidharan et al., 2015). The timestep is maintained with NVT, NPT and MD simulation using the RESPA integer and the whole complex is simulated for 30 ns of MD simulations. The 30 ns MD simulations are analysed for the results from the simulated trajectories using the VMD molecular dynamics visualization tool. The MD simulations are performed for understanding the molecular stability of ligands inside the ion channel and so the results of RMSD values are extracted and the values are plotted (Humphrey et al., 1996).

## 3 Results

### 3.1 Protein-Protein interactions

**Fig. 1:**
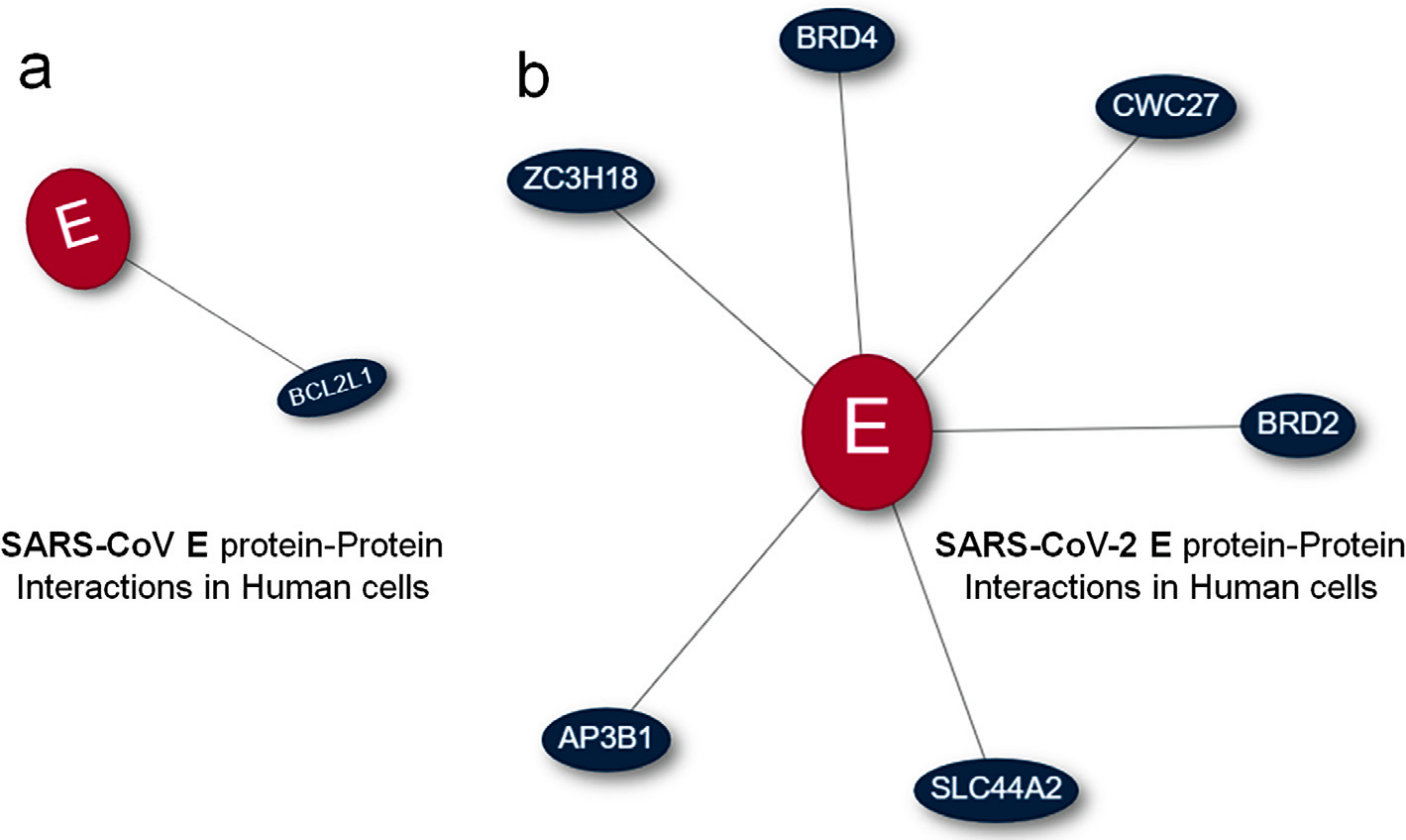
Protein-protein interactions predicted by CoVeX prediction server (a) represents the protein–protein interactions between the SARS-CoV E protein with host protein and (b) represent the protein–protein interactions between the SARS-CoV-2 E protein with host proteins.

The protein–protein interactions are methods to predict the group of protein interactions and here it is applied to understand the host recognition of E protein with both SARS-CoV and SARS-CoV-2. These predictions are related with in vivo experiments, information’s available in literature are applied to calculate the predictions. These interaction predictions help to understand the host proteins interactions with E protein and the results are shown in the Fig. 1. The Fig. 1a shows the results for the protein–protein interactions between the SARS-CoV E protein with host protein and Fig. 1b shows the results for the protein–protein interactions between the SARS-CoV-2 E protein with host protein. The finding clearly states that SARS-CoV E protein interacts with human BCL2L1 protein, but the SARS-CoV-2 E protein interacts with AP3B1, BRD2, BRD4, CWC27, SLC44A2 and ZC3H18 human proteins. These information’s are predicted by the literature and the information’s are provided in Fig. 1.

### 3.2 Multiple sequence alignment

**Fig. 2:**
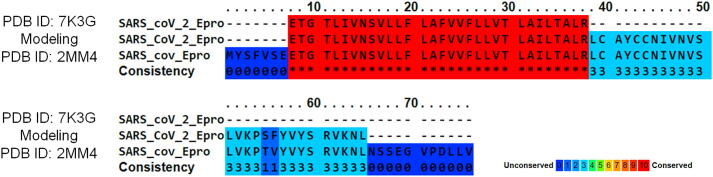
Multiple sequence alignment performed by praline tool for the SARS-CoV E protein (PDB ID 2MM4) along with TMD (PDB ID:7K3G) and full E protein (Modelled) of SARS-CoV-2.

The alignment of three of more proteins or amino acid sequences is said for multiple sequence alignment (MSA) and it provide detail elucidation of pairwise alignments, showing conserved regions with the proteins, which are structural and functionally important. Here the MSA is performed using the praline tool and used to determine the residues aligned as per reference sequences. The results of MSA are provided in the Fig. 2 shows high similarity in the transmembrane domain but there are some changes occurs in the other regions. Here the transmembrane based drug identification will not work, as there is no variation in between the SARS-CoV and SARS-CoV-2 E proteins. Thus, the full sequence structure is required to utilize in purpose of drug design or identifications.

### 3.3 Open and closed state of E protein

Studies suggest that, the E protein in SARS-CoV-2 is having the structural changing phenomenon of open and closed conformations. Recently, the transmembrane conformation domain alone has been solved using the NMR method and available in the protein data bank (PDB ID: 7K3G). On visualization, it clearly shows that the transmembrane domain is the closed state conformation, and for drug design, it will be better to consider the open state conformations. As only the TMD is available, the whole sequence is searched for the suitable templates using blast.

**Fig. 3:**
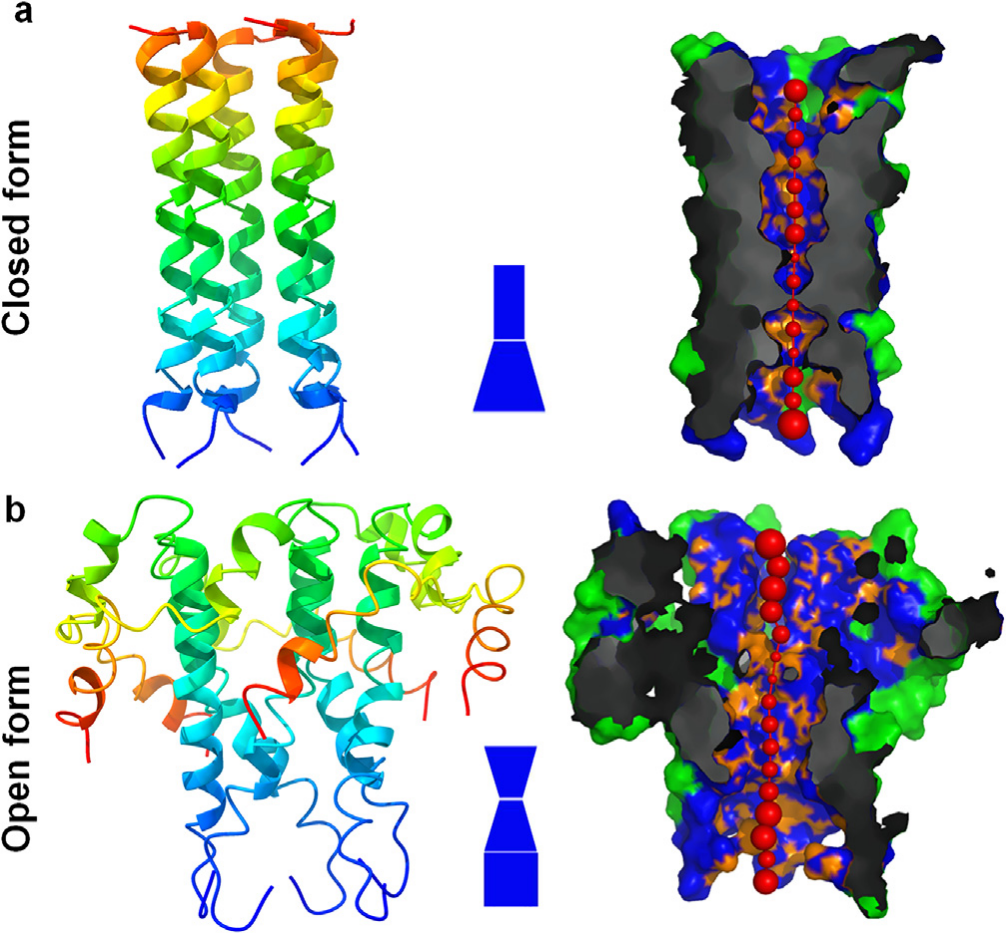
Closed and open state conformations of SARS-CoV-2 E protein shows the pore regions are wider in the open state.

**Fig. 4:**
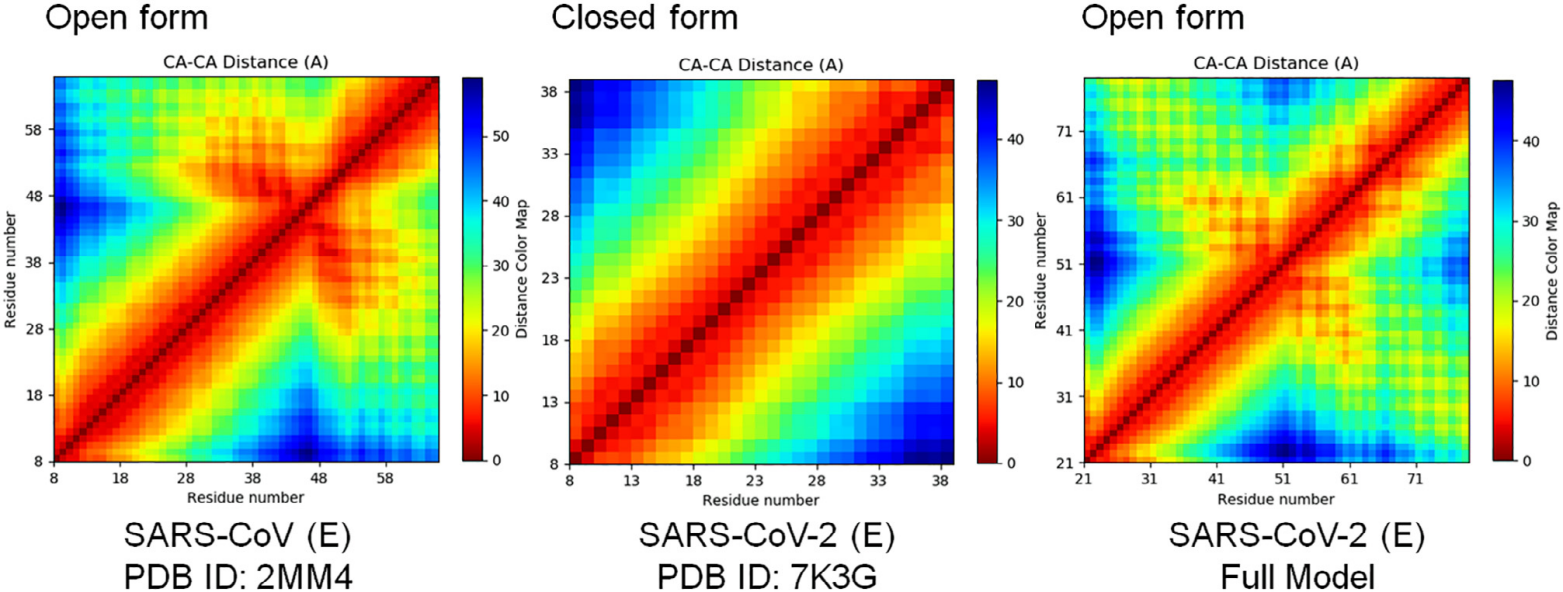
Distance matrix of amino acid for a single chain that tends to make the presence in open and closed chain conformations.

The blast suggests, PDB ID: 2MM4 (E protein of SARS-COV) is chosen as template, considering the open state of the conformation. The model protein looks exactly similar with the template structure and shows the open state conformations. For understanding the structural difference between the open and closed state, the pore walker server is utilized. The pore walker clearly represents the difference between the open and closed state, which is provided in the Fig. 3. The change in conformation of open and closed state may due to the transition states that are applicability in the ion transport mechanism. For understanding the spread of distance between the residues in both open and closed state, the distance matrix is calculated and plotted in the Fig. 4.

From the Fig. 4, it is clear that the open conformations of the E protein in both SARS-CoV and SARS-CoV-2 remain similar, but there are small deviations, that occurs due to the changes in the residue in both viruses. In Fig. 3, the shape of the tunnel is shown in blue for both open and closed state, which clearly shows the wide open in the conformations. When this is compared with results of the distance matrix, the closed conformation of the SARS-CoV-2 E protein is showing narrow red regions. But in case of open state, the distance matrix is wider in few regions in both SARS-CoV and SARS-CoV-2 E protein, as showed in the red regions. Comparing the Fig. 3, Fig. 4 clearly shows the evidence of conformational shift between the open and close state, especially in the transmembrane regions.

### 3.4 Structural Studies on open state E protein in SARS-CoV-2

**Fig. 5:**
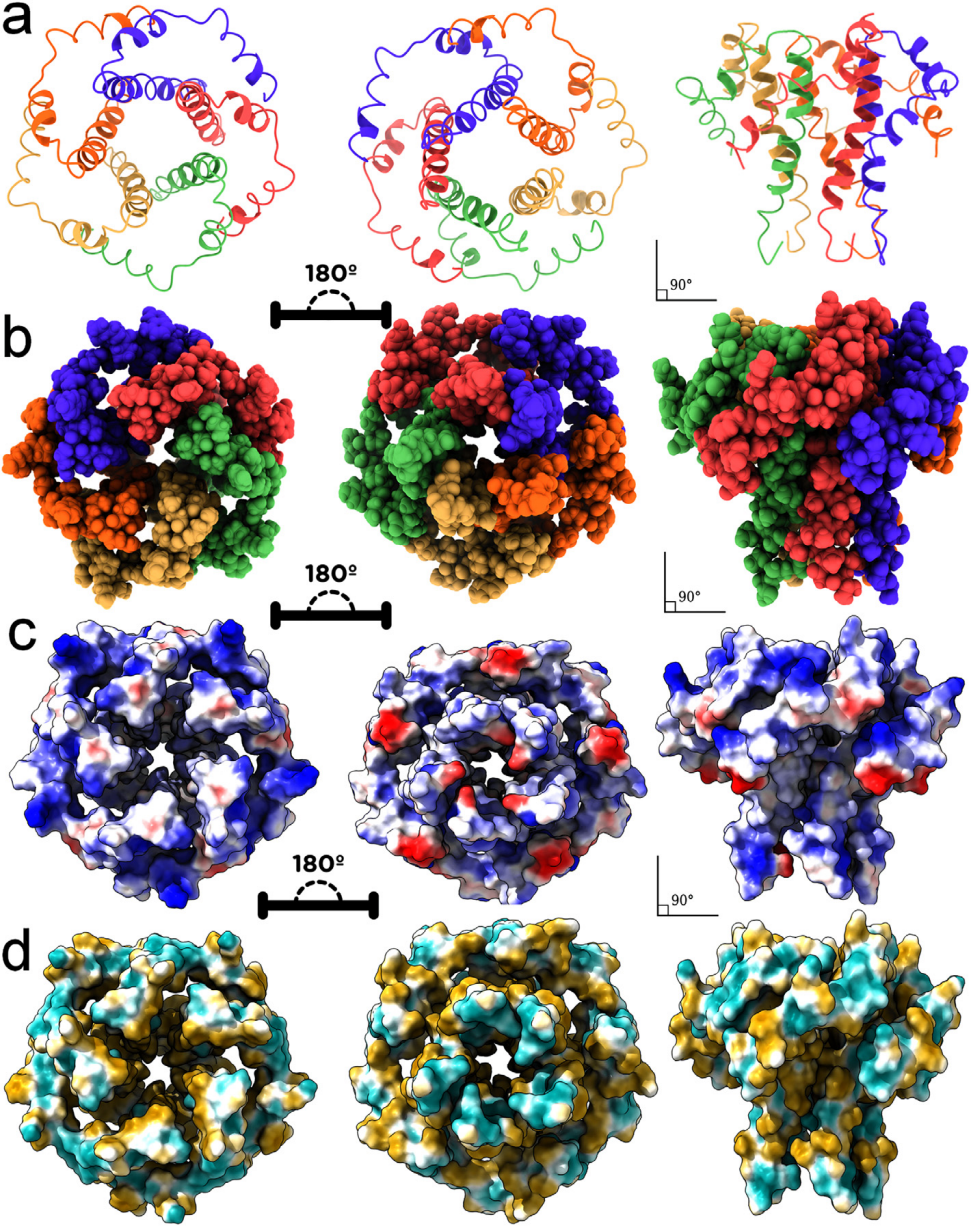
Structural views of SARS-CoV-2 E protein in the open state conformations viewed in (a) cartoonic structure ribbon model, (b) ball mesh without the ribbon model, (c) Electro statistical molecular surface and (d) hydrophobicity surface.

As mentioned, the E protein in SARS-CoV-2 is a membrane integrating ion channel protein that plays the imperial role in budding, the release of progeny viruses from the host cell, and in the activation of host inflammasome. In this, five chains of E protein join to form the homo pentamer, which allows the ion transport with transition change into open and closed state of the enzyme. For detailed understanding of the structure, the homology modelled structure of SARS-CoV-2 E protein in the open state conformations viewed in (Fig. 5a) cartoonic structure ribbon model, (Fig. 5b) ball mesh without the ribbon model, (Fig. 5c) Molecular electrostatic potential surface (MESP) and (Fig. 5d) hydrophobicity surface. The figure provides the clear representation of wide top view, which in 180′ shows narrow funnel shaped, which helps in ion transport as shown in the Fig. 5a and 5b.

Here the colours are indicating each chain and combined to form the homo pentamer structure. The electrostatic potential is analysed for the open state conformation and provided in the Fig. 5c, which shows red coloured spots as negative regions, white coloured spots as neutral regions and blue coloured spots as the positive regions. The results show interesting by showing both positive and negative regions in the outer surface regions, but the pore regions are showing light positive and high neutral regions. But the same pore regions are showing highly hydrophobic as shown in the Fig. 5d, which explains the requirement of hydrophobic ligands for the inhibition of SARS-CoV-2 E protein. For that, the medicinal plant compounds from the *Withania somnifera* is searched in literature and compounds are interacted with pore regions active site.

### 3.5 Molecular docking simulations

Molecular docking is performed for all the compounds from the *Withania somnifera* and each docking is repeated for three times for avoiding the errors. The high-profile compound with low energy values is selectively docked again for getting the final refined conformations in the open state of SARS-CoV-2 E-protein. Final four compounds, which have the binding affinity of lesser energy values than −8.00 are tabulated in the table 1.

**Table 1:** Scoring details of best compounds from *Withania somnifera* interacting with SARS-CoV-2 E protein.

Compound ID	Source from	Binding affinity (kcal/mol)	Amino acid residues	Inhibitor constant
CID 10,100,411	*Withania somnifera*	−9.31	Arg74 (D) Asn77 (D) Val60 (E)	96.94 nM
CID 3,035,439	*Withania somnifera*	−8.98	Arg74 (D) Thr43 (E) Cys46 (E) Ile59 (E)	120.70 nM
CID 101,281,364	*Withania somnifera*	−8.30	Cys46 (E)	13.55 uM
CID 44,423,097	*Withania somnifera*	−8.17	Thr43 (E)	165.81uM

**Fig. 6:**
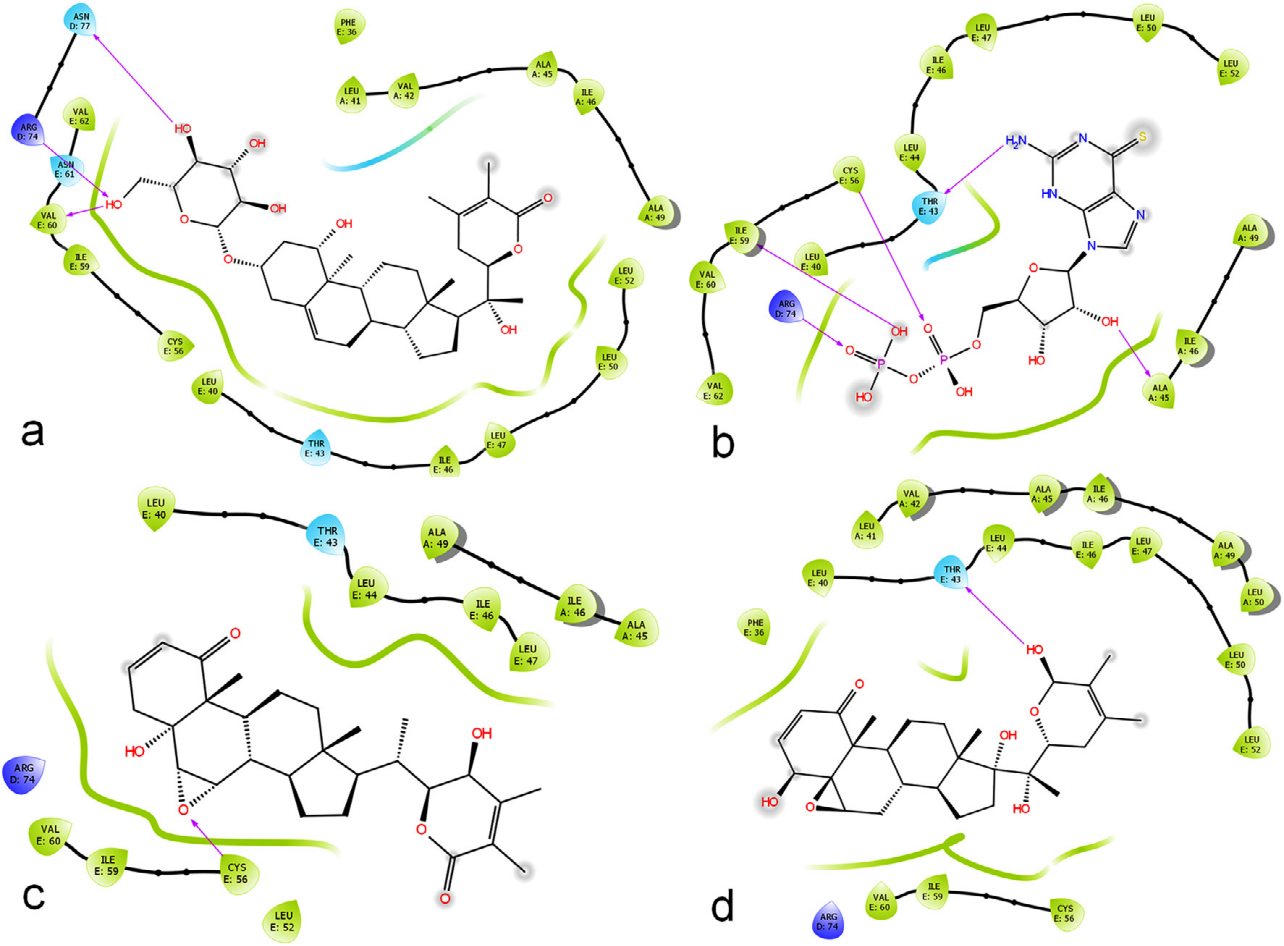
Molecular interactions of compounds namely (a) CID 10100411, (b) CID 3035439, (c) CID 101,281,364 and (d) CID 44,423,097 from Withania somnifera having the tendency to bind with D and E chains of E protein in SARS-CoV-2.

All the best four compounds are having the aromatic ring feature, which are able to bind with hydrophobic regions in the pore regions. While dissecting the interactions, all these four compounds are able to interact with D and E chains of the homo pentamer. All the compounds showing prominent interactions, and 2D interaction viewer showed in Fig. 6 shows, hydrophobic residues surrounded the new leads. On repeating analysis also shows the interactions stronger with the same residues Arg74 (D), Asn77 (D), Thr43 (E), Cys46 (E), Ile59 (E), and Val60 (E). This shows that the above-mentioned residues are having the tendency to interact with ligands and function for the active sites. Final best leads are perfectly found to binding inside the pore region and well interacted with in the pore-lining amino acids. This clearly indicate the inhibitor insights and also the potential active sites residues of Arg74 (D), Asn77 (D), Thr43 (E), Cys46 (E), Ile59 (E), and Val60 (E), which should be targeted for the inhibition of E protein in SARS-CoV-2.

### 3.6 Molecular dynamics simulations

**Fig. 7:**
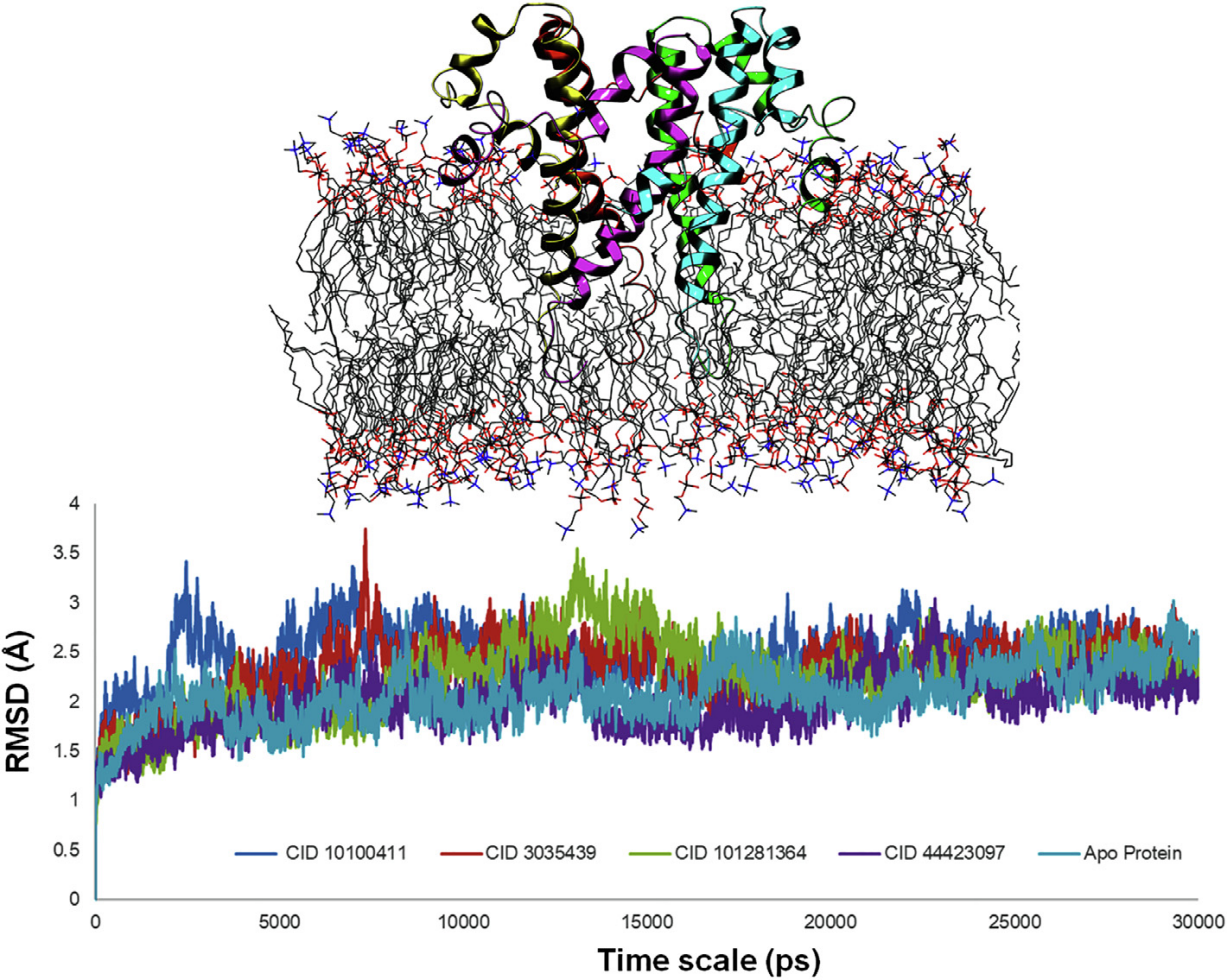
Membrane based MD simulations for both apo and ligand bound E protein complex of SARS-CoV-2 showing stable MD for the 30 ns of timescale.

It is clear that high hydrophobic nature of E protein and integrated with membrane layer and the E protein is placed inside the lipid bilayer membrane as shown in the Fig. 7. For that C terminal is reported to make it presence in the cytoplasm and high hydrophobic N terminal is reported as inserted within the Golgi membrane. So, the membrane is fixed with N-terminal region along with best compounds and simulated for 30 ns. The purpose of the MD simulations is to understand the ligand stability in the dynamic environment along with lipid membrane for providing lively environment and also to understand the dynamic reaction of lead molecules inside the large binding pocket. The Fig. 7 explains the RMSD values plotted with respect to deviations occurs for the apo and ligand bound complex in the membrane. The results show that, the apo and ligand complex are stable inside the membrane-based MD simulations throughout the 30 ns of the MD timescale. Ligands are bit moving due to the pore space, but the amino acids Arg74 (D), Asn77 (D), Thr43 (E), Cys46 (E), Ile59 (E), and Val60 (E) are strongly holding the ligands. For stability, both apo and ligand bound complex lies in between the RMSD values of ~2.2 Å which shows the MD simulations suggest the compounds as best compounds in the dynamic environment.

## 4 Discussion

As of now, there has been several drug targets from both SARS-CoV-2 and host is considered for the drug design and for that, the Spike protein, Main protease and few other proteins are considered with full focus. Along with the above-mentioned proteins, E protein role is imperial and from beginning of the cell entry to exit of cell, E protein plays the major role. The importance of E protein is compared with both SARS-CoV and SARS-CoV-2. Protein-protein interactions based on literature says, that E protein has survival capacity more in SARS-COV-2 than the SARS-COV. This clearly, represent the E protein is an attractive target for drug discovery. Considering this, the work concentrated on describing the structural elucidation of E protein in both closed and open form. This is because, recently the NMR structure of SARS-CoV-2 E protein is solved, but only the domain of transmembrane is solved and that also available in the closed form. Ligand binding modes are not accurate in closed form, as the conformational changes may affect the originality of the ligand interactions. Thus, the work initiated for identifying the open state conformation of the E protein from SARS-COV-2. Initially, the sequence alignment for transmembrane domain is analysed for SARS-COV-2 and SARS-COV, which shows the particular transmembrane domain is exactly matching in between both viruses. But, when the full sequence is compared, the variation among the amino acids is noticeable, and suggest that, for drug identification, only the structure of transmembrane domain is not enough. Based on the MSA analysis, the whole sequence is searched for template and found, the E protein from SARS-CoV is perfectly matched as template and also the template structure possesses the open state conformations. Thus, the same template is considered for homology modelling of open state conformation of SARS-CoV-2 E protein. Multi-chain modelling process with homo pentamer form by combine A-E chains interact together and thus the modelled protein resembles exactly to the template structure. The model protein with open conformation and the NMR solved closed conformations are subject to pore space detection analysis. This shows, the TMD in the closed conformation is narrow, while the open conformation is wide spread which can be considered for the drug design approaches. This is again confirmed with distance matrix calculations, and plotted for each chain. For closed state conformations of SARS-CoV-2, the distance matrix shows narrow profile, but for SARS-CoV and SARS-CoV-2, the distance matrix shows wider profile. But there are few changes seen in between the distance matrix of SARS-CoV and SARS-CoV-2 E protein open state. For understanding the structure of E protein from SARS-CoV-2 in open form is closely visualized for its surface, electrostatic potential surface and hydrophobic surface. These results show interesting by pore surface is light positive charged and highly neutral which also having high hydrophobic surface. This clearly shows the ligand with aromatic pharmacophoric feature can be a better inhibitor, and for that, the medicinal plant-based compounds from *Withania somnifera* is considered for molecular docking analysis. More than 25 compounds are allowed to interact with active sites of SARS-CoV-2 E protein and based on the criteria, the compounds CID 10100411, CID 3035439, CID 101,281,364 and CID 44,423,097 crossed the scrutinization filter by showing lower energy barriers below −8.00 kcal/mol. Interestingly, the best four hit compounds are repeatedly interacting with D and E chains with the amino acids namely, Arg74 (D), Asn77 (D), Thr43 (E), Cys46 (E), Ile59 (E), and Val60 (E). This amino acid tendency is stronger for holding the ligand molecules, that is confirmed by molecular dynamics simulations. For the 30 ns of MD simulations, both apo and ligand complex is stable inside the membrane-based MD simulations. Even though, pore space for the open conformations is widely open in the membrane bound, the amino acids namely, Arg74 (D), Asn77 (D), Thr43 (E), Cys46 (E), Ile59 (E), and Val60 (E) strongly holds the ligand molecules, and keep stable in the dynamic environment.

## 5 Conclusion

Overall, the study suggests the clear molecular understanding of the envelope protein in SARS-CoV-2 for its ion channel activity and given its structural conformational changes, open state conformations are identified. The molecular level understanding of the open state is considered and the hit compounds from from *Withania somnifera* is identified using molecular docking analysis. Through molecular dynamics simulations, the apo and ligand bound complex are stable and recommend CID 10100411, CID 3035439, CID 101,281,364 and CID 44,423,097 these compounds for E protein inhibitors targeting SARS-CoV-2 viral pathogenesis. These compounds, along with other experimental validations, could present themselves as strong potential candidates for SARS-CoV-2.

Ethics approval

Not Applicable

Consent to participate

The author has consent to participate in this manuscript

Consent for publication

The author has consent to publish this manuscript in Saudi journal of Biological Science

Availability of data and material

Data will be available on request to corresponding or first author

Code availability

Not Applicable

## Declaration of Competing Interest

The authors declare that they have no known competing financial interests or personal relationships that could have appeared to influence the work reported in this paper.

## References

[b0005] Altschul S.F., Gish W., Miller W., Myers E.W., Lipman D.J. (1990). Basic local alignment search tool. J Mol Biol.

[b0010] Beema Shafreen R.M., Selvaraj C., Singh S.K., Karutha Pandian S. (2014). In silico and in vitro studies of cinnamaldehyde and their derivatives against LuxS in Streptococcus pyogenes: effects on biofilm and virulence genes. J Mol Recognit.

[b0015] Bianchi M., Benvenuto D., Giovanetti M., Angeletti S., Ciccozzi M., Pascarella S. (2020). Sars-CoV-2 Envelope and Membrane Proteins: Structural Differences Linked to Virus Characteristics?. Biomed Res Int.

[b0020] Chinnasamy S., Selvaraj G., Selvaraj C., Kaushik A.C., Kaliamurthi S., Khan A., Singh S.K., Wei D.Q. (2020). Combining in silico and in vitro approaches to identification of potent inhibitor against phospholipase A2 (PLA2). Int J Biol Macromol.

[b0025] Choudhary P., Bhowmik A., Chakdar H., Khan M.A., Selvaraj C., Singh S.K., Murugan K., Kumar S., Saxena A.K. (2020). Understanding the biological role of PqqB in Pseudomonas stutzeri using molecular dynamics simulation approach. J Biomol Struct Dyn.

[b0030] Dijkstra M.J.J., van der Ploeg A.J., Feenstra K.A., Fokkink W.J., Abeln S., Heringa J. (2019). Tailor-made multiple sequence alignments using the PRALINE 2 alignment toolkit. Bioinformatics.

[b0035] Farag N.S., Breitinger U., Breitinger H.G., El Azizi M.A. (2020). Viroporins and inflammasomes: A key to understand virus-induced inflammation. Int J Biochem Cell Biol.

[b0040] Fazil M.H., Kumar S., Rao N.S., Selvaraj C., Singh S.K., Pandey H.P., Singh D.V. (2012). Comparative structural analysis of two proteins belonging to quorum sensing system in Vibrio cholerae. J Biomol Struct Dyn.

[b0045] Gordon D.E., Jang G.M., Bouhaddou M., Xu J., Obernier K., White K.M., O'Meara M.J., Rezelj V.V., Guo J.Z., Swaney D.L., Tummino T.A., Huttenhain R., Kaake R.M., Richards A.L., Tutuncuoglu B., Foussard H., Batra J., Haas K., Modak M., Kim M., Haas P., Polacco B.J., Braberg H., Fabius J.M., Eckhardt M., Soucheray M., Bennett M.J., Cakir M., McGregor M.J., Li Q., Meyer B., Roesch F., Vallet T., Mac Kain A., Miorin L., Moreno E., Naing Z.Z.C., Zhou Y., Peng S., Shi Y., Zhang Z., Shen W., Kirby I.T., Melnyk J.E., Chorba J.S., Lou K., Dai S.A., Barrio-Hernandez I., Memon D., Hernandez-Armenta C., Lyu J., Mathy C.J.P., Perica T., Pilla K.B., Ganesan S.J., Saltzberg D.J., Rakesh R., Liu X., Rosenthal S.B., Calviello L., Venkataramanan S., Liboy-Lugo J., Lin Y., Huang X.P., Liu Y., Wankowicz S.A., Bohn M., Safari M., Ugur F.S., Koh C., Savar N.S., Tran Q.D., Shengjuler D., Fletcher S.J., O'Neal M.C., Cai Y., Chang J.C.J., Broadhurst D.J., Klippsten S., Sharp P.P., Wenzell N.A., Kuzuoglu-Ozturk D., Wang H.Y., Trenker R., Young J.M., Cavero D.A., Hiatt J., Roth T.L., Rathore U., Subramanian A., Noack J., Hubert M., Stroud R.M., Frankel A.D., Rosenberg O.S., Verba K.A., Agard D.A., Ott M., Emerman M., Jura N., von Zastrow M., Verdin E., Ashworth A., Schwartz O., d'Enfert C., Mukherjee S., Jacobson M., Malik H.S., Fujimori D.G., Ideker T., Craik C.S., Floor S.N., Fraser J.S., Gross J.D., Sali A., Roth B.L., Ruggero D., Taunton J., Kortemme T., Beltrao P., Vignuzzi M., Garcia-Sastre A., Shokat K.M., Shoichet B.K., Krogan N.J. (2020). A SARS-CoV-2 protein interaction map reveals targets for drug repurposing. Nature.

[b0050] Hajjar S.A., Memish Z.A., McIntosh K. (2013). Middle East Respiratory Syndrome Coronavirus (MERS-CoV): a perpetual challenge. Ann Saudi Med.

[b0055] Humphrey W., Dalke A., Schulten K. (1996). VMD: visual molecular dynamics. J Mol Graph.

[b0060] Kumar V., Dhanjal J.K., Bhargava P., Kaul A., Wang J., Zhang H., Kaul S.C., Wadhwa R., Sundar D. (2020). Withanone and Withaferin-A are predicted to interact with transmembrane protease serine 2 (TMPRSS2) and block entry of SARS-CoV-2 into cells. J Biomol Struct Dyn.

[b0065] Loschwitz J., Olubiyi O.O., Hub J.S., Strodel B., Poojari C.S. (2020). Computer simulations of protein-membrane systems. Prog Mol Biol Transl Sci.

[b0070] Mandala V.S., McKay M.J., Shcherbakov A.A., Dregni A.J., Kolocouris A., Hong M. (2020). Structure and drug binding of the SARS-CoV-2 envelope protein transmembrane domain in lipid bilayers. Nat Struct Mol Biol.

[b0075] Mukherjee S., Bhattacharyya D., Bhunia A. (2020). Host-membrane interacting interface of the SARS coronavirus envelope protein: Immense functional potential of C-terminal domain. Biophys Chem.

[b0080] Muralidharan A.R., Selvaraj C., Singh S.K., Sheu J.R., Thomas P.A., Geraldine P. (2015). Structure-Based Virtual Screening and Biological Evaluation of a Calpain Inhibitor for Prevention of Selenite-Induced Cataractogenesis in an in Vitro System. J Chem Inf Model.

[b0085] Nayarisseri A., Khandelwal R., Madhavi M., Selvaraj C., Panwar U., Sharma K., Hussain T., Singh S.K. (2020). Shape-based Machine Learning Models for the Potential Novel COVID-19 Protease Inhibitors Assisted by Molecular Dynamics Simulation. Curr Top Med Chem.

[b0090] Paital B. (2020). Nurture to nature via COVID-19, a self-regenerating environmental strategy of environment in global context. Sci Total Environ.

[b0095] Pfefferle S., Schopf J., Kogl M., Friedel C.C., Muller M.A., Carbajo-Lozoya J., Stellberger T., von Dall'Armi E., Herzog P., Kallies S., Niemeyer D., Ditt V., Kuri T., Zust R., Pumpor K., Hilgenfeld R., Schwarz F., Zimmer R., Steffen I., Weber F., Thiel V., Herrler G., Thiel H.J., Schwegmann-Wessels C., Pohlmann S., Haas J., Drosten C., von Brunn A. (2011). The SARS-coronavirus-host interactome: identification of cyclophilins as target for pan-coronavirus inhibitors. PLoS Pathog.

[b0100] Sadegh S., Matschinske J., Blumenthal D.B., Galindez G., Kacprowski T., List M., Nasirigerdeh R., Oubounyt M., Pichlmair A., Rose T.D., Salgado-Albarran M., Spath J., Stukalov A., Wenke N.K., Yuan K., Pauling J.K., Baumbach J. (2020). Exploring the SARS-CoV-2 virus-host-drug interactome for drug repurposing. Nat Commun.

[b0105] Satarker S., Nampoothiri M. (2020). Structural Proteins in Severe Acute Respiratory Syndrome Coronavirus-2. Arch Med Res.

[b0110] Selvaraj C., Dinesh D.C., Panwar U., Abhirami R., Boura E., Singh S.K. (2020). Structure-based virtual screening and molecular dynamics simulation of SARS-CoV-2 Guanine-N7 methyltransferase (nsp14) for identifying antiviral inhibitors against COVID-19. J Biomol Struct Dyn.

[b0115] Selvaraj, C., Dinesh, D. C., Panwar, U., Boura, E., Singh, S. K., 2020b. High-Throughput Screening and Quantum Mechanics for Identifying Potent Inhibitors against Mac1 Domain of SARS-CoV-2 Nsp3. IEEE/ACM Trans Comput Biol Bioinform PP. 10.1109/TCBB.2020.3037136 PMC8769010 33306471

[b0120] Selvaraj C., Omer A., Singh P., Singh S.K. (2015). Molecular insights of protein contour recognition with ligand pharmacophoric sites through combinatorial library design and MD simulation in validating HTLV-1 PR inhibitors. Mol Biosyst.

[b0125] Selvaraj C., Panwar U., Dinesh D.C., Boura E., Singh P., Dubey V.K., Singh S.K. (2020). Microsecond MD Simulation and Multiple-Conformation Virtual Screening to Identify Potential Anti-COVID-19 Inhibitors Against SARS-CoV-2 Main Protease. Front Chem.

[b0130] Selvaraj C., Sakkiah S., Tong W., Hong H. (2018). Molecular dynamics simulations and applications in computational toxicology and nanotoxicology. Food Chem Toxicol.

[b0135] Selvaraj C., Singh P., Singh S.K. (2014). Molecular insights on analogs of HIV PR inhibitors toward HTLV-1 PR through QM/MM interactions and molecular dynamics studies: comparative structure analysis of wild and mutant HTLV-1 PR. J Mol Recognit.

[b0140] Shah V.K., Firmal P., Alam A., Ganguly D., Chattopadhyay S. (2020). Overview of Immune Response During SARS-CoV-2 Infection: Lessons From the Past. Front Immunol.

[b0145] Sivakamavalli J., Selvaraj C., Singh S.K., Vaseeharan B. (2014). Exploration of protein-protein interaction effects on alpha-2-macroglobulin in an inhibition of serine protease through gene expression and molecular simulations studies. J Biomol Struct Dyn.

[b0150] Tang T., Bidon M., Jaimes J.A., Whittaker G.R., Daniel S. (2020). Coronavirus membrane fusion mechanism offers a potential target for antiviral development. Antiviral Res.

[b0155] Umesh, Kundu, D., Selvaraj, C., Singh, S. K., Dubey, V. K., 2020. Identification of new anti-nCoV drug chemical compounds from Indian spices exploiting SARS-CoV-2 main protease as target. J Biomol Struct Dyn, 1-9. 10.1080/07391102.2020.1763202 PMC7232883 32362243

[b0160] Yadav S., Gupta S., Selvaraj C., Doharey P.K., Verma A., Singh S.K., Saxena J.K. (2014). In silico and in vitro studies on the protein-protein interactions between Brugia malayi immunomodulatory protein calreticulin and human C1q. PLoS One.

